# Uterine Torsion due to Sigmoid Colon Volvulus in a Pregnant Woman: A Case Report

**DOI:** 10.1002/ccr3.70854

**Published:** 2025-09-04

**Authors:** Nooshin Abasi, Zahra Javanbakht, Keyvan Karimabadi, Mohammad Hossein Golezar, Setareh Soltani, Tahereh Parsajam

**Affiliations:** ^1^ Department of Obstetrics & Gynecology Motazedi Hospital, Kermanshah University of Medical Sciences Kermanshah Iran; ^2^ School of Medicine Kermanshah University of Medical Sciences Kermanshah Iran; ^3^ Advanced Diagnostic and Interventional Radiology Research Center (ADIR), Medical Imaging Center Tehran University of Medical Sciences Tehran Iran; ^4^ Clinical Research Development Center Taleghani and Imam Ali Hospital, Kermanshah University of Medical Sciences Kermanshah Iran

**Keywords:** colectomy, pregnancy, sigmoid volvulus, uterus torsion

## Abstract

Sigmoid volvulus and uterine torsion are both rare and challenging conditions in pregnancy, and the coexistence of these conditions is particularly difficult to diagnose. Herein, we report a case of a 38‐year‐old pregnant woman at 30 weeks of gestation, with a history of two prior cesarean sections, who presented with severe abdominal pain, vomiting, and constipation, and was eventually diagnosed with both sigmoid volvulus and uterine torsion during surgery. Clinicians should consider the possibility of bowel obstruction when a pregnant woman presents with severe abdominal pain, vomiting, and constipation, as early diagnosis is crucial. Early imaging, prompt intervention, and a multidisciplinary approach are essential for preventing severe maternal and fetal complications.

**Summary**

- Severe abdominal pain with vomiting and constipation should prompt evaluation for bowel obstruction, not be dismissed as common pregnancy symptoms.
- Early imaging, with at least an abdominal X‐ray, and a multidisciplinary approach are vital to reduce maternal and fetal morbidity and mortality in pregnant patients presented with bowel obstruction symptoms.

## 1 Introduction

In obstetric practice, uterine torsion during pregnancy is an uncommon complication, described as the uterus rotating more than 45° around its longitudinal axis [1]. The complications of uterine torsion in pregnancy are primarily determined by the degree of rotation, gestational age, and the time elapsed between the onset of symptoms and treatment [2]. Although the underlying cause of uterine torsion remains unclear, various risk factors have been identified, including morphologic defects, pelvic tumors, uterine fibroids, adhesions, traction forces, and aberrant fetal presentation [3].

Sigmoid volvulus is a very uncommon condition in pregnancy. It typically presents as bowel obstruction, but physiological changes of pregnancy, such as abdominal pain, nausea, and leukocytosis, can mislead clinicians and delay appropriate diagnostic evaluation. Sigmoid volvulus has a relatively poor prognosis during pregnancy, with maternal mortality ranging from 6% to 21% and fetal mortality from 26% to 50% [4, 5, 6].

In this case, by presenting a patient with both sigmoid volvulus and uterine torsion, which together make it an exceptionally rare condition, we aim to describe its nature, its negative impact on the fetus and mother, and the importance of early diagnosis and intervention.

## 2 Case History/Examination

A 38‐year‐old female patient, gravida 3, para 2, with a history of two prior cesarean sections and a gestational age of 30 weeks and 1 day, presented to the hospital with severe abdominal pain, vomiting, abdominal distension, and failure to defecate. The patient had been experiencing progressive severe abdominal pain, anorexia, nausea, vomiting, and constipation for 1 week before hospital presentation. The vomitus contained food material. Her first cesarean section was performed due to 5 years of infertility and an unsuccessful in vitro fertilization attempt. Initially, she had made appointments and had been visited by a gynecologist and a gastroenterologist at outpatient clinics, where she was treated and managed outside the hospital. However, due to a lack of improvement and worsening symptoms, she presented to the hospital 2 days before the operation. Given the absence of contractions, a negative tocometry, and stable vital signs, she was discharged with her personal consent and a prescription for cefixime. The following day, she returned with worsening symptoms and was subsequently hospitalized. She was afebrile and presented with tachycardia and hypotension, with initial vital signs including a blood pressure of 90/60 mmHg, a pulse of 110 beats per minute, and a respiratory rate of 18 breaths per minute. Abdominal examination revealed severe distension, generalized tenderness, and rebound tenderness. Due to the severe abdominal pain, the fundus of the uterus was difficult to palpate. Tocometry showed moderate contractions with an interval of 7–8 min.

## 3 Differential Diagnosis and Investigations

Although an erect abdominal X‐ray might be considered safe, both X‐ray radiography and computed tomography (CT) imaging were avoided prior to the operation. No nasogastric tube was inserted. Laboratory tests revealed a hemoglobin level of 10.1 g/dL, a leukocyte count of 17.9 × 10^9^/L, and a normal platelet count of 229 × 10^9^/L, with prothrombin time, partial thromboplastin time, international normalized ratio, serum creatinine, serum urea, aspartate transaminase, alanine transaminase, and bilirubin all within the normal range. The patient experienced contractions every 15 min, with an interval of 7–8 min, and was monitored using a nonstress test and tocodynamometer. Fetal heart rate was not detected at 8:00 PM, and at 9:00 PM, she was transferred to the operating room with a diagnosis of intrauterine fetal demise (IUFD) and underwent a cesarean section.

## 4 Outcome and Follow‐Up

**FIGURE 1:**
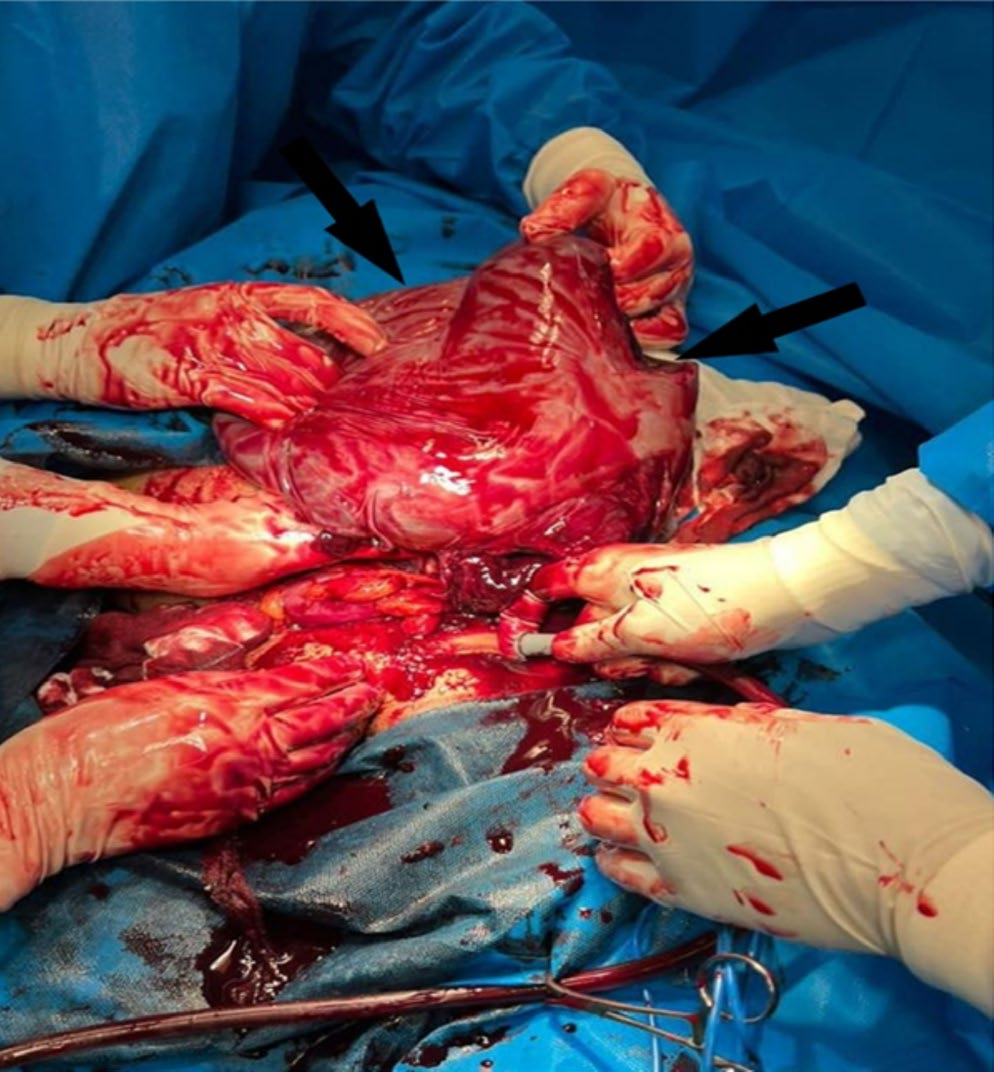
Intraoperative view of the uterus showing incision from the posterior.

**FIGURE 2:**
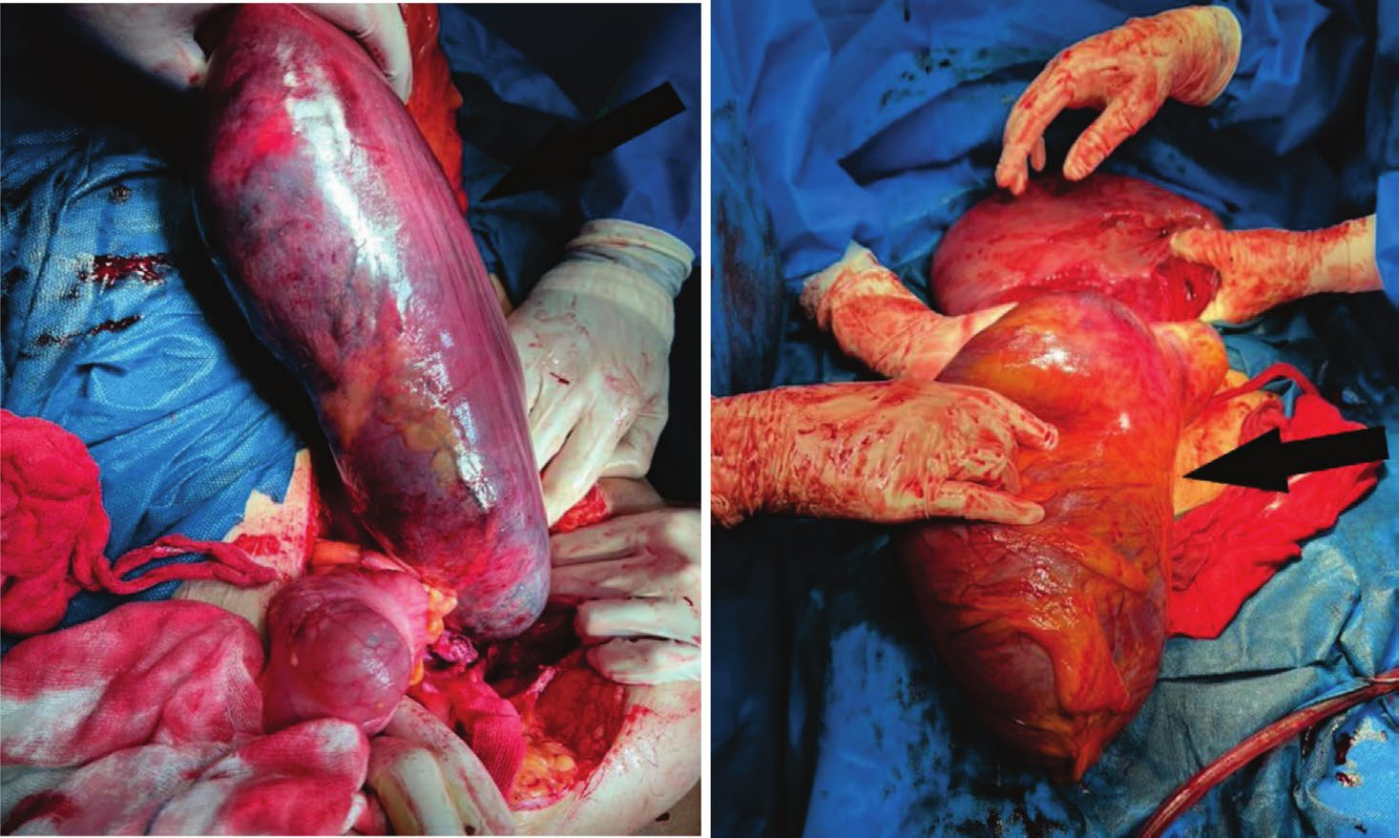
Intraoperative views of sigmoid colon showing dilation and ischemia of the colon.

At first inspection, the abdomen appeared extremely edematous and distended. A Pfannenstiel incision was made in the anatomical direction, and upon opening the abdomen, a large amount of pus was expelled. The IUFD fetus was then removed, followed by the placenta, which contained multiple clots. The uterus was completely pulpy and had numerous hematomas (Figure 1). Uterine torsion was observed, with the uterus having rotated completely for 180° clockwise, along with necrosis of the sigmoid colon and part of the descending colon. The bilateral round ligaments were identified to avoid an incorrect uterine incision. The incision for fetal removal was made at the posterior of the uterus. There was no adhesion between the uterus and the sigmoid colon, and the abundance of pus inside the abdomen was attributed to the necrosis of the sigmoid colon. After the cesarean section and fetal removal, the patient underwent a total hysterectomy and right oophorectomy due to extensive gangrene of the uterus. The decision for hysterectomy was made after consultation with a multidisciplinary team. According to the surgical report, uterine torsion and ischemia of the sigmoid colon were evident, leading to the surgical resection of the sigmoid colon and the proximal part of the rectum (Figure 2). The abdomen was then irrigated, and a corrugated drain was placed. The patient subsequently underwent a Hartmann's colostomy. According to the urology service's evaluation, there was extensive damage to the right ureter, which was cut proximally at the site of the mesocolon during the colectomy procedure. Due to the inability to perform a closed nephrostomy, the patient became a candidate for an open nephrostomy in the pelvis. The patient was finally discharged after 54 days of hospitalization. The nephrostomy was removed, but the colostomy remained in place for more than one year.

## 5 Discussion

Pregnancy, particularly in the third trimester when over 70% of cases occur, appears to increase the risk of sigmoid volvulus due to the displacement of a mobile sigmoid colon by the enlarging uterus [7, 8]. This anatomical alteration, combined with pregnancy‐related physiological changes that may slow gastrointestinal motility and lead to constipation, increases the risk of volvulus [8, 9]. About 10% of pregnant patients with sigmoid volvulus, including our case, have a history of prior abdominal surgeries, most commonly cesarean sections, which often lead to adhesions in the lower abdomen and pelvic region [8]. In our case, after opening the abdomen and removing the IUFD, it was evident that the uterus had torn open at the back, caused by the sigmoid volvulus. Based on the sequence of symptoms, which began with vomiting and constipation, it is likely that the rotation of the sigmoid colon occurred first and subsequently led to uterine torsion, highlighting the interdependence of these two rare complications.

Uterine torsion and sigmoid volvulus are both uncommon in pregnancy, and the coexistence of these two conditions in a single case is exceptionally rare. To our knowledge, this is the second reported case of both conditions occurring together, the first being reported by Tehrani et al. in 2013 in a 30‐year‐old pregnant woman [10]. This rarity complicates diagnosis, as the clinical presentation often overlaps with more common pregnancy‐related complications, such as preterm labor, placental abruption, or acute appendicitis [10]. In addition, uterine rupture should always be considered in the presence of sudden abdominal pain and an abnormal fetal heart rate [11]. The clinical ambiguity is further exacerbated by the reluctance to perform radiographic imaging during pregnancy due to concerns about fetal exposure to ionizing radiation, even though these imaging modalities can significantly aid in diagnosis [8]. While ultrasound is frequently used as the initial imaging modality in pregnancy, it has limited utility in diagnosing volvulus and uterine torsion. Therefore, a comprehensive clinical examination is required when managing a patient with similar symptoms.

Currently, the preferred diagnostic method for sigmoid volvulus is a CT scan of the abdomen, which can confirm the diagnosis with a specificity of more than 90% and a sensitivity of nearly 100% in the nonpregnant population [12]. However, the absence of timely imaging in pregnant patients often results in a diagnosis only being made intraoperatively.

The management of sigmoid volvulus and uterine torsion in pregnancy requires a multidisciplinary approach involving obstetricians, surgeons, and anesthesiologists [13]. Timely intervention is essential to minimize maternal and fetal morbidity and mortality. A recently published systematic review highlighted that endoscopic detorsion, in cases with isolated sigmoid or cecal volvulus, can significantly improve fetal survival [8]. In our case, the advanced necrosis of the sigmoid colon and ischemia of the uterus necessitated extensive surgical intervention, including hysterectomy and right oophorectomy, in addition to colectomy. These procedures highlight the severity of complications that can arise from delayed diagnosis [14, 15].

The maternal complications of uterine torsion include hemodynamic shock and complete or partial placental abruption. On the other hand, fetal complications often involve hypoxia, antepartum hemorrhage, and the risk of IUFD [1, 16], which, unfortunately, occurred in our case. These adverse outcomes emphasize the importance of early recognition and timely intervention. Given the rarity and high‐risk nature of these conditions, a high index of suspicion should be maintained in pregnant women presenting with unexplained abdominal pain and symptoms of intestinal obstruction, such as distension, vomiting, or failure to pass stool. Early imaging, particularly CT when appropriate, should be considered in cases with ambiguous clinical presentations.

In conclusion, this case highlights the exceptional rarity and complexity of concurrent sigmoid volvulus and uterine torsion during pregnancy. Managing such a complex case needs a multidisciplinary approach to improve outcomes for both the mother and fetus.

## Author Contributions

**Nooshin Abasi:** investigation, methodology, project administration, resources, supervision, writing – original draft. **Zahra Javanbakht:** methodology, resources, supervision, writing – original draft. **Keyvan Karimabadi:** investigation, visualization, writing – original draft. **Mohammad Hossein Golezar:** methodology, resources, supervision, writing – original draft, writing – review and editing. **Setareh Soltani:** project administration, supervision, writing – review and editing. **Tahereh Parsajam:** resources, visualization, writing – original draft.

## Ethics Statement

All ethical and moral issues have been considered in this study.

## Consent

Written informed consent was obtained from the patient to publish this report in accordance with the journal's patient consent policy.

## Conflicts of Interest

The authors declare no conflicts of interest.

## Data Availability

The data that support the findings of this study are available from the corresponding author upon reasonable request.
